# Effects of heavy metal exposure on kidney transplant recipients: mechanisms and clinical implications for graft failure risk

**DOI:** 10.3389/fimmu.2025.1718695

**Published:** 2025-11-28

**Authors:** Shuxin Li, Hongliang Cao, Binbin Wang, Gengchen Huang, Yongliang Qu, Bo Yuan, Wei Wei

**Affiliations:** 1 Department of Urology, The First Hospital of Jilin University, Changchun, China; 2 Organ Transplant Center, First Hospital of Jilin University, Changchun, China

**Keywords:** heavy metals, kidney transplant, environmental pollution, graft failure, oxidative stress

## Abstract

Environmental exposure to heavy metals, such as cadmium, lead, arsenic, and copper, represents a significant yet underappreciated threat to the long-term survival of kidney transplants. Accumulating epidemiological evidence consistently links even low-level exposure to a substantially elevated risk of late graft failure. The transplanted kidney is particularly vulnerable due to its heightened susceptibility to oxidative stress, compounded by immunosuppressive therapy and often impaired excretory function. The core pathophysiological mechanism involves the accumulation of heavy metals in the renal cortex, where they disrupt mitochondrial function and catalyze the generation of reactive oxygen species (ROS) via Fenton-like reactions. This oxidative surge depletes antioxidant defenses, triggering a deleterious cascade of inflammation, apoptosis, and fibrosis, which accelerates the progression of chronic allograft injury. Recognizing this modifiable environmental risk factor is paramount for improving outcomes. This review synthesizes the current evidence and proposes a multi-pronged management strategy, encompassing rigorous biomonitoring, targeted dietary interventions, and novel therapeutic approaches, such as mitochondrial transplantation and the use of natural antioxidants, to mitigate heavy metal toxicity and enhance graft longevity.

## 1 Introduction

With the rapid advancement of global industrialization and urbanization, heavy metal pollutants pose an increasingly severe threat to ecological environments and human health. Heavy metals such as cadmium, lead, mercury, and arsenic exhibit high toxicity, environmental persistence, non-biodegradability, and bioaccumulation (1–3). They resist degradation in natural environments yet accumulate continuously through food chains, thereby causing long-term harm to ecosystems and human health (1). These pollutants primarily originate from human activities, including mining, smelting, industrial wastewater discharge, pesticide application, electronic waste disposal, and various emissions generated during urbanization (4–6). This leads to their widespread distribution in soil, water bodies, and air (3, 6, 7). Heavy metals enter the environment through multiple pathways, including wastewater, exhaust gases, and solid waste. They not only accumulate near their emission points but also migrate to previously uncontaminated areas, forming complex pollution and exacerbating ecological risks (8–10). The accumulation of heavy metals is particularly pronounced in coastal waters, lake sediments, and urban environments, severely impacting the environmental balance and sustainable human habitats (11–13). When humans ingest heavy metals through contaminated air, drinking water, or the food chain, these elements gradually accumulate in the body (7, 14, 15). This accumulation can trigger multi-system health damage, including neurological disorders, kidney failure, immune system dysfunction, digestive system disorders, skin diseases, reproductive abnormalities, respiratory diseases, and potential carcinogenic risks (14–16). Long-term exposure to heavy metals, even at low concentrations, may cause progressive, irreversible damage to multiple organs, posing a significant global public health challenge.

The kidneys serve as the body’s primary excretory and metabolic organs. Due to their high blood flow and the reabsorption and concentration functions of the renal tubules, they are more prone to accumulating heavy metal elements, making them a key target for heavy metal toxicity (17–19). Consequently, they become key targets for heavy metal toxicity. After entering the kidneys via the bloodstream, heavy metals exert nephrotoxic effects through multiple mechanisms, including induction of oxidative stress, mitochondrial dysfunction, inflammatory responses, apoptosis, and direct damage to renal tubular epithelial cells (20–24). Chronic low-level exposure to heavy metals and the resulting subclinical kidney injury have become a focal point in public health. Such damage often presents insidiously in its early stages yet exhibits persistent progression, significantly increasing the risk of chronic kidney disease and end-stage renal disease while severely compromising renal transplant outcomes (25–28). Therefore, against the backdrop of widespread heavy metal contamination, elucidating the nephrotoxic mechanisms of these metals is crucial for developing early biomarkers, identifying high-risk populations, and formulating targeted prevention strategies. This review summarizes the sources and exposure pathways of several major heavy metal pollutants, with a focus on their core molecular mechanisms that induce nephrotoxicity. It aims to provide theoretical foundations for toxicity prevention research and to reduce the risk of kidney transplant failure.

## 2 Epidemiological evidence indicates a strong association between heavy metal exposure and kidney transplant failure

Recent epidemiological studies have identified a significant association between heavy metal exposure and kidney transplant failure. Environmental exposure to heavy metals poses a serious threat to kidney graft function, substantially increasing the risk of graft failure and renal decline. Recognizing heavy metal exposure as a controllable risk factor in the long-term management of transplanted kidneys and implementing corresponding exposure prevention strategies holds significant potential clinical implications for improving graft survival.

### 2.1 Increased risk of graft failure associated with heavy metal exposure

Heavy metals are ubiquitous in the environment and workplace, and their nephrotoxicity is widely recognized as a potential contributor to the onset and progression of chronic kidney disease (CKD). In recent years, epidemiological research has increasingly focused on the impact of heavy metal exposure on the specific population of kidney transplant recipients (KTRs), particularly its association with graft failure risk. Multiple prospective cohort studies indicate that even at levels typical of environmental exposure, heavy metals significantly increase the risk of graft failure in KTRs. A Dutch prospective cohort study suggested that a doubling of plasma lead concentration in KTRs was associated with a 59% increase in the risk of late graft failure, implying that controlling lead exposure may represent a novel approach to improving long-term graft survival (29). This finding remained significant after multivariable adjustment for major clinical and cardiovascular risk factors, confirming lead exposure as an independent risk factor (29). Separately, a study of 693 KTRs found that doubling urinary copper excretion increased the risk of transplant failure by 57%, demonstrating a dose-response relationship. This mechanism may relate to copper-induced oxidative damage to renal tubules (30). Additionally, elevated plasma cadmium concentrations were independently associated with long-term graft failure and declining renal function (31). Similarly, among 665 KTRs, plasma arsenic concentrations were independently associated with an 80% increased risk of late graft failure, with fish consumption identified as the primary source of arsenic exposure (32). In summary, existing epidemiological evidence consistently indicates that even low-level environmental heavy metal exposure, which is conventionally considered safe, is significantly associated with an increased risk of long-term graft failure in KTRs (33, 34). These findings are consistent with a causal relationship between heavy metal exposure and graft failure and highlight environmental heavy metal exposure as a critical, potentially modifiable risk factor for graft failure in KTRs. However, definitive confirmation of causality awaits support from intervention studies or quasi-experimental evidence. Although prospective cohort studies control major confounding factors through methods such as multivariable adjustment and reveal exposure-response relationships of public health significance, caution is still needed in causal inferences. Potential residual confounding, exposure misclassification, time-varying exposures, and competing risks may still affect the accuracy of effect estimates.

### 2.2 Summary of epidemiological evidence on heavy metal exposure and kidney transplant failure

Existing epidemiological evidence consistently indicates that even low-level environmental heavy metal exposure is significantly associated with an increased risk of long-term graft failure in KTRs. However, while current studies reveal these associations, they also expose several key issues. First, low-dose, long-term cumulative, and mixed exposures to multiple heavy metals represent the main realistic risk scenarios faced by transplant populations, and their combined toxic effects may be more complex. Second, exposure levels measured in biological matrices, such as blood or urine, may differ from the actual heavy metal load in transplant kidney tissue. Additionally, observational studies cannot entirely rule out reverse causation, in which declining kidney function reduces heavy metal excretion, leading to elevated blood concentrations. These factors limit the causal inference of the exposure-outcome relationship. Therefore, it is urgently necessary to elucidate the exact molecular pathways of heavy metal-induced nephrotoxicity and, on this basis, to conduct targeted interventional studies to consolidate the causal chain of this association and provide a solid foundation for the development of subsequent clinical prevention strategies.

## 3 Mechanisms by which heavy metal exposure affects kidney transplant outcomes

Although current observational studies have revealed an association between heavy metal exposure and adverse outcomes in kidney transplantation, and these studies’ differences in characterization provide targeted strategies for prevention and treatment, the specific mechanisms of action require further clarification. This section will elaborate on the potential mechanisms linking heavy metal exposure to kidney transplant failure and how such exposure influences the development of transplanted kidneys. The complex relationship between heavy metal exposure and adverse kidney transplant outcomes involves multiple pathways, including nephrotoxic effects, oxidative stress, and cumulative effects.

### 3.1 Heavy metal accumulation and specific vulnerabilities in transplanted organs

Heavy metals such as cadmium, lead, and arsenic exhibit a significant affinity for the kidneys (35). Following environmental or occupational exposure, they preferentially accumulate in the renal cortex (35). By inducing oxidative stress and mitochondrial dysfunction, they directly damage renal tubular epithelial cells and glomerular structures (31, 36). This accumulation phenomenon also occurs in transplanted kidneys, and because the kidneys are frequently hyper filtrated, the toxic effects may be further amplified. Even when circulating heavy metal concentrations remain within conventional detection ranges, their long-term accumulation in transplanted kidney tissue can persistently induce oxidative damage (29, 37). When multiple heavy metals co-expose, they may synergistically activate oxidative stress pathways, thereby accelerating the progression of renal injury (38, 39). Transplant recipients constitute a specialized group exhibiting heightened sensitivity to heavy metal toxicity. Long-term immunosuppressive therapy weakens the body’s antioxidant defenses, making transplanted kidneys more susceptible to oxidative stress attacks (34, 40). Additionally, certain immunomodulatory drugs may interfere with heavy metal metabolism or inhibit renal self-repair mechanisms, thereby increasing susceptibility to toxicity (37). Although total heavy metal accumulation in transplanted kidneys may be lower than in kidneys removed due to tumors, their intrinsic compensatory mechanisms against heavy metal toxicity are significantly weakened by prolonged subclinical inflammation (37). Transplanted kidneys often carry preexisting pathological foundations from ischemia-reperfusion injury or rejection-related conditions. Heavy metal exposure can further exacerbate oxidative damage, breaching physiological compensation thresholds (31, 40, 41). Moreover, with partial functional impairment in transplanted kidneys, the efficiency of heavy metal excretion decreases, leading to higher accumulation levels compared to normal kidneys (42–44). This accelerates the progression of renal fibrosis and chronic transplant nephropathy.

Exposure to heavy metals in the environment typically occurs as mixtures, which may produce additive or interactive effects when inducing nephrotoxicity (38, 39). For example, cadmium and arsenic may synergistically inhibit the Nrf2 antioxidant pathway, making cells more susceptible to oxidative damage (45, 46). However, the role of Nrf2 in heavy metal-induced nephrotoxicity is complex and dual. While chronic low-dose exposure to heavy metals is generally associated with Nrf2 suppression, recent evidence suggests that acute or high-intensity exposure may trigger persistent overactivation of Nrf2 signaling (47, 48). This abnormal sustained activation is not protective; it may lead to harmful consequences, including the induction of autophagy blockage and a vicious cycle of oxidative stress-autophagy inhibition, further weakening the cell’s antioxidant defenses (47, 48). Such overactivation can cause ‘transcriptional exhaustion,’ meaning that after long-term efforts to combat oxidative stress, the cell can no longer maintain subsequent antioxidant gene expression, leading to the collapse of the antioxidant defense system and autophagy dysfunction (47, 48). Moreover, persistent activation of Nrf2 can reprogram cellular metabolism and promote the expression of profibrotic factors, directly exacerbating renal tubular interstitial inflammation and fibrosis (49). This mechanism involves Nrf2 interfering with normal cellular signaling pathways, which under pathological conditions can transform into a pro-fibrotic effect (49). In the specific context of transplanted kidneys, the inherent oxidative stress state interacts with heavy metal exposure, making the Nrf2 pathway more prone to shift from early protective activation to decompensated dysfunction (50, 51). And lead and copper may have cumulative effects at the mitochondrial level, jointly aggravating disturbances in the electron transport chain and increasing ROS bursts (52). This complex interaction makes risk assessments based on a single metal likely to underestimate the actual health risk. Therefore, in future mechanistic and epidemiological studies, the use of advanced statistical models, such as weighted quantile regression and Bayesian kernel machine regression, is crucial for clarifying the overall effects of mixed heavy metal exposure on transplant kidney injury, identifying key driving components, and understanding potential interactions (53, 54).

### 3.2 Oxidative stress

**Figure 1:**
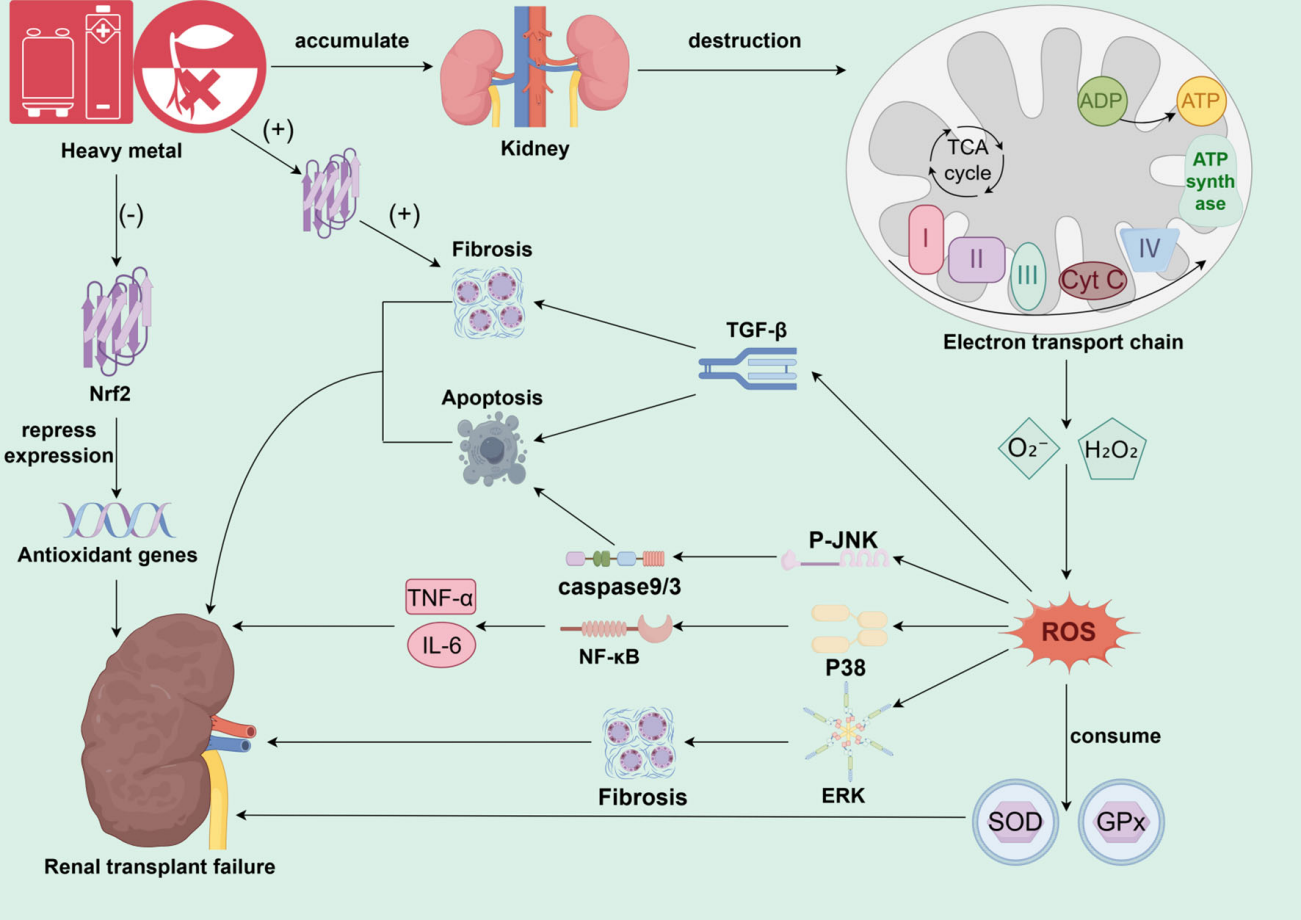
The core mechanism of heavy metal-induced oxidative stress and chronic injury in the transplanted kidney. This figure illustrates the core molecular pathways involved in environmental heavy metal exposure leading to transplanted kidney failure. Upon accumulation of heavy metals in the renal cortex, the primary target is to disrupt renal tubular mitochondrial function, inducing a burst of ROS generation by interfering with the electron transport chain and catalyzing the Fenton reaction. The dramatically elevated ROS disrupts cellular redox homeostasis, depletes endogenous antioxidants such as GSH, and inhibits key antioxidant enzymes, such as SOD and GPx, on the one hand. On the other hand, it leads to a comprehensive dysregulation of cellular antioxidant defense system by inhibiting or abnormally activating the Nrf2 pathway. Dysregulation of the cellular antioxidant defense system. Sustained oxidative damage triggers direct cellular damage such as lipid peroxidation, protein modification, and DNA breakage, and in turn activates three key MAPK signaling pathways (JNK, p38, Erk), which dominate apoptosis, inflammation, and fibrosis, respectively. Meanwhile, ROS also activate the NF-κB inflammatory pathway and TGF-β pro-fibrotic pathway. These pathways are intertwined, and together they lead to renal tubular epithelial cell death, chronic inflammation and interstitial fibrosis, which ultimately drive the progression of chronic transplant kidney injury until the loss of transplant kidney function.

Heavy metals are ubiquitous in the environment and occupational settings, entering the human body through multiple pathways and specifically accumulating in the renal cortex, thereby inducing significant nephrotoxic effects. Their core toxic mechanism is closely associated with the induction of oxidative stress (**Figure 1**). Heavy metals can disrupt the mitochondrial electron transport chain in renal tubules, leading to an increased production of superoxide anion (O_2_^-^) and hydrogen peroxide (H_2_O_2_) (55–58). Metal ions catalyze the Fenton reaction to generate large amounts of reactive oxygen species (ROS) (52, 59, 60). This process disrupts intracellular redox balance, continuously depleting endogenous antioxidants, such as glutathione, and directly inhibits the activity of key antioxidant enzymes, including superoxide dismutase (SOD) and glutathione peroxidase (GPx) (45, 58, 61). Furthermore, heavy metal exposure inhibits the activation of nuclear factor E2-related factor 2 (Nrf2), thereby obstructing the expression of downstream antioxidant genes (45, 46). In transplanted kidneys, heavy metal-induced ROS surges further lead to the depletion of antioxidant enzymes and the accumulation of lipid peroxides, directly damaging renal cell membrane structures and compromising DNA integrity (31, 38, 39, 43, 62). Oxidative damage ultimately triggers cellular harm, including lipid peroxidation, protein modification, and DNA breaks. This oxidative stress response not only directly damages tubular epithelial cells and glomerular structures, causing acute or chronic kidney injury, but also further activates inflammatory signaling pathways, including NF-κB and MAPK (60, 63, 64).In addition, different members of the mitogen-activated protein kinase (MAPK) signaling pathway play specific roles in heavy metal-associated kidney injury. This family mainly includes extracellular signal-regulated kinase (Erk), c-Jun N-terminal kinase (JNK), and p38 MAPK. The Erk pathway exhibits a dual function in heavy metal nephrotoxicity: while it primarily participates in cell proliferation and survival under physiological conditions, its sustained abnormal activation under heavy metal stress may mediate excessive proliferation and phenotypic transformation of intrinsic kidney cells, thereby promoting the progression of renal interstitial fibrosis (65, 66). Specifically, uranium can promote proliferation signals by enhancing Erk phosphorylation, whereas chromium inhibits its activity, weakening the cell’s self-repair ability; using an Erk inhibitor can effectively alleviate cadmium-triggered inflammatory responses (67–69). In contrast, the JNK and p38 pathways are generally considered core mediators of stress responses and are highly sensitive to oxidative stress caused by heavy metals. The JNK pathway primarily regulates apoptosis. Metals like cadmium and uranium induce a burst of reactive oxygen species (ROS), significantly increasing the p-JNK/JNK ratio, thereby activating the mitochondrial apoptosis pathway and leading to renal tubular epithelial cell death through a caspase-9/3 cascade reaction (67, 70–72). The p38 pathway primarily governs the initiation and amplification of inflammatory responses. Cadmium and lead promote p38 phosphorylation, further activating NF-κB and the NLRP3 inflammasome, leading to massive release of key inflammatory factors such as TNF-α and IL-1β (60, 69). Moreover, p38 can form a positive feedback loop with signaling pathways like endoplasmic reticulum stress, synergistically aggravating tissue damage (60, 73). It is noteworthy that there is close interaction among these MAPK sub-pathways. For instance, cadmium and chromium can jointly activate JNK and p38, synergistically enhancing apoptotic signals (72, 74).Different heavy metals also show pathway preferences, such as lead being more inclined to activate JNK (75). Upstream oxidative stress events collectively regulate all these pathways. ROS induced by cadmium, chromium, uranium, and other metals can directly phosphorylate and activate Erk, JNK, and p38, forming a core ROS-MAPK signaling axis (76). In summary, under heavy metal exposure, a sharp increase in ROS can simultaneously or preferentially activate distinct MAPK signaling pathways. This promotes the release of pro-inflammatory factors, such as TNF-α and IL-6, which drive localized chronic inflammation in the transplanted kidney and amplify its effects (60, 63, 64). Concurrently, ROS activates the caspase-dependent apoptosis pathway and induces TGF-β expression, leading to tubular epithelial cell apoptosis and subsequent interstitial fibrosis (31, 42, 58). In summary, oxidative stress-mediated cellular injury not only disrupts functional recovery in transplanted kidneys but may also contribute to acute rejection. Studies confirm that post-transplant oxidative stress levels serve as key biomarkers for acute rejection, inducing renal cell inflammation and necrosis that delay graft functional recovery or cause long-term dysfunction.

### 3.3 Genetic susceptibility

Beyond the direct pathophysiological mechanisms, individual genetic susceptibility plays a pivotal role in modulating the risk and severity of heavy metal nephrotoxicity in KTRs. Genetic polymorphisms can influence an individual’s ability to metabolize, detoxify, and excrete heavy metals, as well as the resilience of renal tissue to the ensuing oxidative stress and inflammation (77). As highlighted by Glicklich et al. in their review on heavy metal toxicity in chronic kidney disease and cardiovascular disease, genetic variations are key determinants of inter-individual differences in toxicant handling and disease manifestation (77). This principle is highly relevant to the transplant population. Variations in genes involved in the glutathione (GSH) synthesis and conjugation pathways, such as glutathione S-transferases (GSTs), can significantly impact cellular antioxidant capacity and the elimination of heavy metals, thereby modifying the extent of oxidative damage (78, 79). Specific polymorphisms also illustrate gene-environment interactions that heighten susceptibility to renal injury. The AA genotype of rs13244925 in the epidermal growth factor receptor (EGFR) gene has been associated with higher estimated glomerular filtration rate (eGFR) in cadmium-exposed individuals, suggesting a protective role against cadmium nephrotoxicity that is absent in non-exposed groups (80). Similarly, single-nucleotide polymorphisms (SNPs) in the tumor necrosis factor-α (TNF-α) gene increase susceptibility to metal-induced CKD by upregulating transmembrane TNF-α expression, particularly worsening renal injury through chronic inflammation and fibrosis in advanced CKD (81). Polymorphisms in the vascular endothelial growth factor A (VEGFA) gene exacerbate the risk of renal dysfunction under co-exposure to lead and cadmium (81). In both additive and recessive genetic models, these SNPs—combined with urinary cadmium and blood lead levels—significantly modulate renal dysfunction risk, underscoring their role in altering baseline renal function and amplifying heavy metal toxicity. Furthermore, variants in inflammation-related genes, such as NLRP3, enhance fibrosis propensity in response to metal exposure, significantly elevating CKD risk by amplifying chronic inflammatory responses (82). Certain allelic combinations exert multiplicative effects, further increasing the risk of graft failure and highlighting how genetic variants promote susceptibility via renal interstitial fibrotic pathways. In kidney transplantation, this genetic predisposition interacts with the allograft’s unique milieu. The recipient’s genetic background controls systemic metal metabolism and immune-inflammatory responses, whereas the donor’s genetic makeup influences the kidney’s intrinsic resilience after transplantation. The two interact in a complex manner that ultimately determines the outcome of grafts under heavy metal exposure. Therefore, omitting genetic susceptibility from risk assessments may lead to an incomplete understanding and underestimation of threats faced by vulnerable KTR subpopulations.

## 4 Potential prevention and treatment strategies

**Figure 2:**
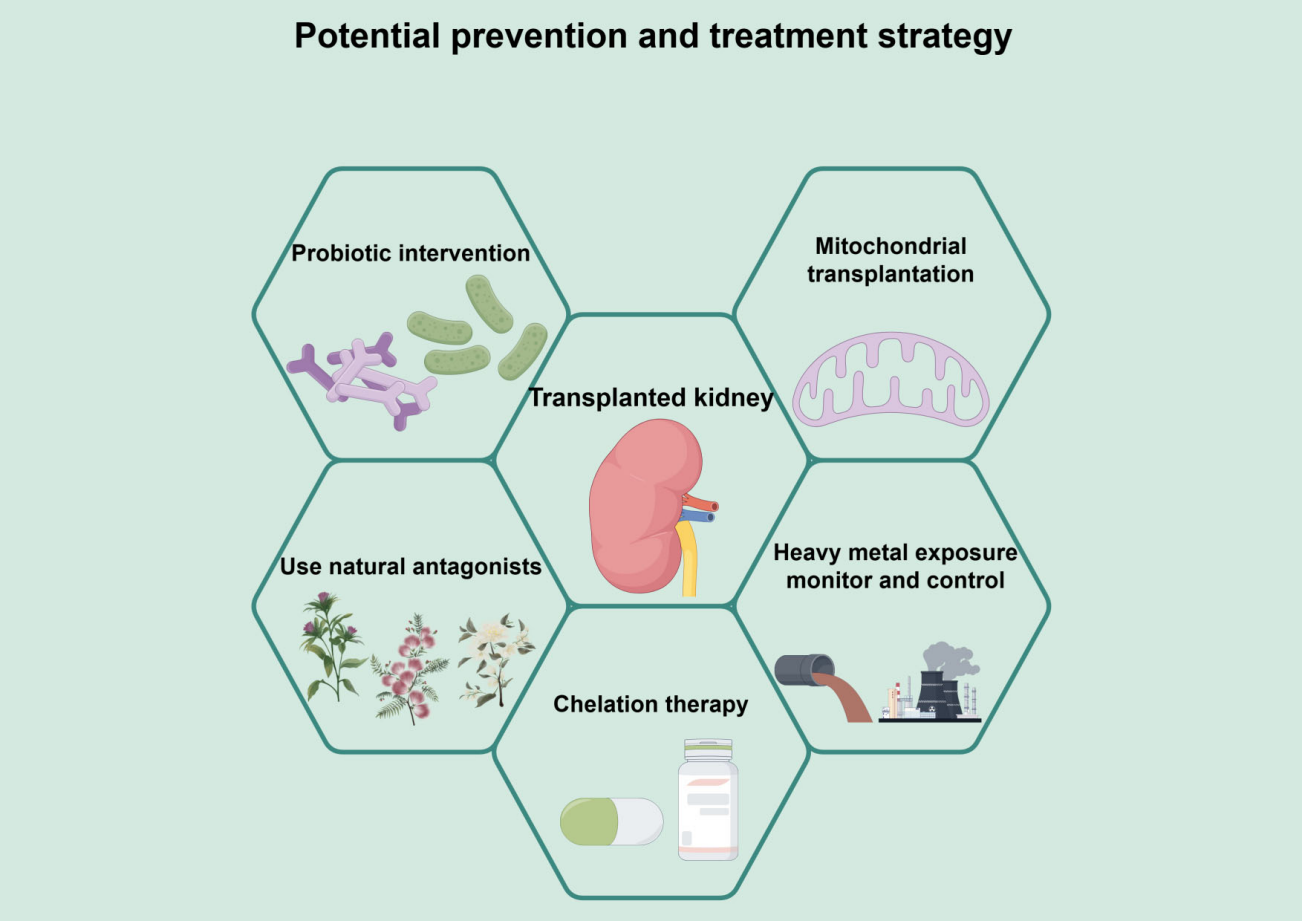
A multi-pronged management framework for mitigating heavy metal-associated injury in kidney transplant recipients. This figure presents a framework for a comprehensive intervention strategy to mitigate heavy metal toxicity and protect kidney function after transplantation. The framework is based on environmental control and biomonitoring to limit exposure through source reduction and early warning. On this basis, multiple intervention strategies are integrated. Mitochondrial transplantation can directly repair heavy metal-induced mitochondrial dysfunction and restore energy metabolism from the root. Probiotic intervention reduces the bioavailability of heavy metals through adsorption and chelation, enhances systemic antioxidant capacity, and improves intestinal barrier function through the intestinal-renal axis. Natural antagonists activate endogenous antioxidant pathways, such as Nrf-2/HO-1, and inhibit inflammation, thereby improving the function of transplanted kidneys. Natural antagonists activate endogenous antioxidant pathways such as Nrf-2/HO-1, which in turn inhibit inflammatory and apoptotic signaling. Chelation therapy may be considered to promote heavy metal excretion after cautious evaluation of patients with clearly excessive body burdens. These strategies target the key components of heavy metal toxicity - mitochondrial damage, intestinal absorption, oxidative stress, and body load - synergistically, and work together to mitigate secondary damage, such as inflammation and fibrosis, to decrease heavy metal accumulation, alleviating oxidative stress, and thus enhancing renal transplantation function.

To reduce the risk of graft failure in KTR due to heavy metal exposure, the researchers proposed a set of multi-level integrated intervention strategies (**Figure 2**). The system is based on environmental control and biomonitoring to reduce exposure at the source and achieve early warning. Based on this system, mitochondrial transplantation to restore cellular energy metabolism, probiotic interventions to minimize absorption and enhance antioxidant defense through the intestinal-renal axis, and natural antagonists to activate the endogenous antioxidant pathway are employed to address the key aspects of heavy metal-induced mitochondrial damage, oxidative stress, and intestinal absorption, thus synergizing to reduce secondary damage such as inflammation and fibrosis. It is important to note that several of the proposed strategies—namely mitochondrial transplantation, probiotic supplementation, and the use of natural antagonists—currently rest largely on preclinical evidence derived from animal studies and *in vitro* models. Although they represent promising therapeutic avenues, their safety, efficacy, and practical feasibility in the immunocompromised KTR population remain to be validated in future clinical trials. The following sections detail the mechanistic rationale and experimental support for each strategy, and address the challenges associated with their clinical translation.

### 4.1 Mitochondrial transplantation

To address the risk of renal toxicity and transplant failure associated with heavy metal exposure, mitochondrial transplantation has emerged as a promising but experimental therapeutic strategy in preclinical research. Heavy metals primarily damage renal tubes by inducing oxidative stress, disrupting mitochondrial function, and triggering apoptosis, thereby significantly increasing the risk of graft loss (83–85). Further research indicates that heavy metals specifically damage highly metabolically active mitochondria in the kidney, disrupting their energy supply and waste-clearance capabilities, thereby accelerating post-transplant renal deterioration (42, 44, 85). Mitochondrial transplantation directly repairs heavy metal-induced mitochondrial dysfunction by exogenously supplying healthy mitochondria, effectively reversing oxidative stress and cellular damage (86–89). Preclinical studies in a cadmium-induced nephrotoxicity model have shown that this therapy significantly elevated intracellular reduced glutathione levels, enhanced Bcl-2 anti-apoptotic protein expression, and inhibited caspase-3 activity (86). This promoted ATP synthesis recovery and renal tubular structural regeneration, improving renal function indicators and reducing histopathological damage (86–88). Additionally, mitochondrial transplantation mitigates immunosuppressant-related mitochondrial toxicity and alleviates postoperative oxidative stress, thereby contributing to a comprehensive reduction in the risk of graft dysfunction (90, 91). Although still far from clinical application, mitochondrial transplantation theoretically offers a novel intervention approach in kidney transplantation. However, the promising evidence for mitochondrial transplantation currently derives primarily from preclinical animal studies and *in vitro* models. Its clinical translation faces several bottlenecks, including the standardized sourcing of viable mitochondria, the risk of immune reactions to allogeneic organelles, the development of safe and efficient *in vivo* delivery methods to the kidneys, and navigating the substantial regulatory and ethical hurdles associated with such a novel cellular therapy.

### 4.2 Probiotic intervention

Experimental research, primarily in animal models, indicates that probiotics, such as Lactobacillus and Bacillus, can significantly reduce markers of oxidative damage in kidney tissue and restore antioxidant enzyme activity through their antioxidant properties. For instance, probiotic supplementation after nickel exposure upregulates protective gene expression in the kidneys, reversing the suppression of gene expression caused by heavy metals (63). This antioxidant effect is particularly crucial for immunosuppressed kidneys post-transplantation, mitigating secondary oxidative damage (63). Heavy metal exposure disrupts gut microbiota, exacerbating renal burden through the leaky gut effect. Probiotics directly reduce intestinal heavy metal absorption through mechanisms such as biosorption and bioaccumulation, while repairing intestinal barrier functions to block the migration of inflammatory factors to the kidneys (92–94). Clinical studies in occupationally exposed populations demonstrate that probiotic-containing yogurt reduces urinary cadmium levels by 60%-72% in occupationally exposed populations, while promoting the proliferation of the beneficial gut bacterium Bifidobacterium (95, 96). The specific probiotic strain *Lactobacillus acidophilus* exhibits a high-affinity binding capacity for cadmium, mercury, and other heavy metals, converting them into less toxic forms for excretion via surface proteins or metabolic enzymes (96–98). For instance, probiotic strains isolated from kimchi significantly reduced renal cadmium accumulation and alleviated tubular necrosis in mice (97). This targeted detoxification reduces heavy metal burden in transplanted kidneys. Moreover, probiotics modulate the Th1/Th2 immune balance to mitigate chronic inflammation induced by heavy metals (92, 99). In KTRs, this immunoregulation may help mitigate synergistic damage from transplant rejection and heavy metal toxicity. Furthermore, synergistic supplementation with probiotics and minerals, such as calcium/iron can further reduce cadmium bioavailability, offering novel strategies for post-transplant nutritional support (94). In summary, preclinical evidence suggests that probiotics may serve as an adjunctive therapy to mitigate the risks of heavy metal exposure in transplanted kidneys through multi-pathway actions involving intestinal detoxification, systemic antioxidant effects, and renal protection (92, 100). While the efficacy of probiotics against heavy metals is supported by studies in occupationally exposed and general populations, direct evidence in KTRs is lacking. Critical safety considerations in this immunocompromised cohort include the risk of probiotic-derived infections and potential interactions with immunosuppressive drugs. Future research should prioritize randomized feasibility trials with endpoints focusing on probiotic tolerability, alongside pharmacokinetic monitoring of immunosuppressants and indicators of metal excretion.

### 4.3 The use of natural antagonists

Exposure to heavy metals constitutes a significant risk factor for graft failure in KTRs, with its toxic effects primarily mediated through inducing oxidative stress and precipitating renal cellular damage (31, 34). Natural antagonists, including plant extracts, polyphenols, and hormonal substances, have shown potential in experimental settings to alleviate oxidative stress and delay the decline in transplanted kidney function by activating endogenous antioxidant pathways. For instance, melatonin not only suppresses the production of reactive oxygen species (ROS) and enhances superoxide dismutase (SOD) activity, but also reduces heavy metal accumulation in the kidneys, thereby exerting renal protective effects (101). Polyphenolic extracts derived from seaweed improve renal function indicators by directly counteracting heavy metal-induced oxidative stress (102). Furthermore, the compound OOP isolated from traditional Chinese medicine has demonstrated pivotal value in preventing and treating transplant-associated renal injury by inhibiting tubular epithelial cell apoptosis and mitigating oxidative damage through the activation of the Nrf2/HO-1 signaling pathway (103). The mechanisms of action for these natural compounds include enhancing systemic antioxidant capacity and directly chelating heavy metals to reduce their bioavailability, thereby achieving multi-pathway renal protection (104, 105). At the therapeutic strategy level, natural antagonists have demonstrated efficacy in preclinical models. For instance, certain plant extracts, acting as potent antioxidants, significantly alleviate cadmium-induced oxidative stress and renal structural damage. Modulating key signaling pathways further amplifies antioxidant responses, offering potential intervention targets for renal transplant recipients (45, 104, 106). Despite bioavailability limitations, experimental delivery strategies, such as nanomedicines, are being explored in early-stage research to enhance their *in vivo* efficacy and applicability (107, 108). In summary, natural antagonists demonstrate promising prospects for counteracting heavy metal-related transplanted kidney injury through their multi-mechanism synergistic effects. The translation of natural antagonists is constrained by questions regarding optimal dosing, bioavailability of conventional formulations, and their intricate pharmacokinetic profiles. The development of novel delivery systems, such as nanomedicines, must therefore prioritize the validation of their safety profile and exclusion of any adverse interference with the transplant pharmacotherapy.

However, when considering any interventional strategy to promote heavy metal excretion, a critical clinical paradox must be acknowledged. The excretion process itself may transiently increase the renal toxicant load, potentially exacerbating kidney injury (109). This is particularly evident in the treatment of cadmium poisoning. Although chelating agents are conventional methods for cadmium removal, clinical observations indicate that mobilization of cadmium from storage sites during treatment can lead to a temporary surge in blood and renal cadmium concentrations (110). This surge may precipitate a wave of “secondary injury” in individuals with pre-existing renal impairment (111). Therefore, for KTRs, a vulnerable group with limited functional reserve and whose transplanted kidneys often exhibit subclinical injury, any proactive detoxification treatment must be approached with great caution. Future research should focus on developing gentler, more controlled detoxification strategies and on incorporating close monitoring of early kidney injury biomarkers during treatment. The paramount principle must be the absolute priority of protecting graft function throughout the detoxification process.

### 4.4 Chelation therapy

For KTRs whose body loads of specific heavy metals have been definitively exceeded, chelation therapy is a way to reduce toxic metal loads through medication directly. As comprehensively reviewed by Glicklich and Frishman, there is a strong case for screening for heavy metal exposure in at-risk populations, including those with chronic kidney disease, as elevated levels of heavy metals are associated with a poorer prognosis (112). Chelating agents (e.g., EDTA) act by forming stable, water-soluble complexes with metal ions in the bloodstream, thereby promoting their excretion through the urine (110, 112, 113). Evidence from studies of non-transplanted patients with chronic kidney disease and confirmed lead toxicity suggests that chelation therapy improves renal function and slows disease progression (77, 112). This provides a rationale for considering this therapy in selected KTRs. However, the application of chelation therapy in KTRs requires extreme caution due to the vulnerability of transplanted organs. The process of mobilizing metals from tissue stores may temporarily increase the concentration of nephrotoxic substances flowing through the renal tubules, thus posing a risk of “secondary injury” to the graft (110, 114, 115). Transplanted kidneys, which often have a reduced functional reserve and are already subclinical compromised, may be particularly vulnerable to such damage. Therefore, the decision to use chelation therapy for KTR must be individualized and implemented only after careful risk-benefit assessment. It should be limited to those cases where there is clear evidence that high heavy metal loads are associated with progressive decline in graft function and where environmental exposures have been clearly controlled. Treatment must begin at low doses and be administered under close clinical supervision with strict monitoring of graft function, electrolytes, and renal injury biomarkers. Collaboration between transplant nephrologists and clinical toxicologists is essential to navigate this complex therapeutic area. Future studies are needed to develop specific safety and efficacy protocols for chelation therapy for the KTR population.

### 4.5 Environmental heavy metal exposure control and monitoring

Environmental heavy metal exposure is widely recognized as a significant risk factor for kidney damage, particularly for KTRs, where even exposure levels within the normal range may increase the risk of graft failure. Research indicates that heavy metal nephrotoxicity arises from their accumulation in renal tissue following environmental exposure, triggering oxidative stress and inflammatory responses that reduce glomerular filtration rate and lead to graft dysfunction (31, 42, 116). Prospective cohort analyses demonstrate that elevated plasma cadmium levels are significantly associated with an increased risk of kidney transplant failure, independent of other clinical factors, with a progressive increase in risk corresponding to longer exposure duration (31). In the general population, mixed heavy metal exposure may also accelerate chronic kidney injury progression, posing greater threats to transplant recipients with pre-existing renal impairment (53, 116, 117). Environmental exposure control is a core strategy for mitigating risks. This includes reducing heavy metal concentrations in ecological media through regulating pollution sources and implementing environmental protection policies. Strict management of drinking water sources, air particulate matter, and soil contaminants can significantly lower ingestion exposure risks. Research confirms that air pollution exposure is significantly and positively correlated with mortality, graft failure, and rejection risks among KTRs, underscoring the importance of pollution control as a modifiable environmental risk factor (117–119). Furthermore, assessing total environmental exposure levels is crucial for developing targeted interventions, such as using biomarkers to monitor heavy metal concentrations or conducting exposure assessments in polluted areas (120–122). Concurrently, monitoring exposure levels forms the foundation of risk management. Systematic monitoring involves real-time evaluation of heavy metal concentrations in environmental and biological samples to identify high-risk populations and regions. For instance, epidemiological studies recommend using urinary cadmium and lead levels as exposure indicators, combined with early biomarkers of kidney injury, to enable stratified monitoring of susceptibility in KTRs (53, 116, 123, 124). This monitoring not only aids in identifying exposure sources but also supports the development of combined exposure risk models to predict graft failure trends and optimize prevention strategies (54, 122). Finally, comprehensive prevention measures must integrate control and monitoring, including reducing individual exposure, strengthening occupational and community regulations, and promoting mechanism-based interventions such as drug development targeting metal excretion pathways (44, 122). Future efforts should involve multidisciplinary collaboration to optimize pollution management, thereby reducing the burden of heavy metal-related nephrotoxicity and improving long-term outcomes in kidney transplantation.

## 5 Clinical management stratification and process recommendations

Given the risks above, it is crucial to integrate management of environmental heavy metal exposure into the long-term follow-up system for KTRs, and a stratified management strategy is recommended. For high-risk recipients living in highly polluted areas or engaged in related occupations, baseline assessments should be conducted upon enrollment. Exposure biomarkers, such as urinary cadmium and blood lead, should be monitored regularly every 6–12 months during follow-up, along with early kidney injury markers, including kidney injury molecule-1, neutrophil gelatinase-associated lipocalin, and β2-microglobulin, to capture subclinical toxicity sensitively (125–127). Early indicators of heavy metal-induced kidney injury are key to identifying this injury and predicting graft outcomes in KTRs. Oxidative stress-related biomarkers, such as 8-hydroxy-2’-deoxyguanosine (8-OHdG), protein carbonyls, and the redox ratio (GSH/GSSG), can directly reflect free-radical damage induced by heavy metal exposure (128, 129). Elevated urinary 8-OHdG is closely associated with heavy metal exposure and, as a reliable marker of DNA oxidative damage, can be used to assess the risk of early subclinical kidney injury (128, 129). F2-isoprostanes and oxidized albumin profiling enhance the monitoring of lipid peroxidation and protein modification, supplementing the prediction of tubular interstitial injury. Meanwhile, quantifying inflammatory cytokine profiles, such as IL-6 and TNF-α, can reveal chronic inflammatory responses triggered by heavy metals, which are related to graft rejection and long-term functional decline (34, 130). The combined use of these indicators, such as KIM-1 and NGAL, has been shown to effectively distinguish between exposed and non-exposed groups, providing early warning signals (128, 131, 132). Regarding lifestyle, specific guidance should be provided, including reducing the intake of seafood known to be high in heavy metals, being aware of heavy metals contamination risks in rice and drinking water, and supplementing sufficient minerals such as selenium and calcium to reduce intestinal absorption of heavy metals through competitive inhibition (133–138). When monitoring indicates elevated exposure levels, a structured intervention pathway should be initiated. First, potential sources of exposure in personal life and work environments should be actively investigated. Then, supportive measures can be taken, such as nutritional interventions, probiotic supplementation, or the use of natural antioxidants. Additionally, consider consulting with occupational or environmental health experts to identify and control source exposure. Throughout the process, the most critical aspect is maintaining close coordination with the core management of the transplant team, ensuring that any interventions do not compromise blood concentrations or the efficacy of immunosuppressive drugs, thereby reducing heavy metal toxicity while safeguarding the transplanted kidney’s long-term survival.

## 6 Future research directions

Future research should focus on deepening our understanding of the association between heavy metal exposure and kidney transplant outcomes across multiple levels, while advancing clinical translation. At the mechanistic level, multi-omics technologies should be employed to elucidate interactions among core pathways within the unique microenvironment of transplanted kidneys, such as oxidative stress, inflammation, and fibrosis, with particular emphasis on the amplified effects of these processes under immunosuppression. Regarding exposure assessment and risk prediction, establish comprehensive risk models for mixed heavy metal exposure using large-scale prospective cohorts, integrating factors such as genetic susceptibility and immune status to enable precise risk stratification. Simultaneously develop novel biomarkers capable of early detection of heavy metal accumulation and subclinical injury within transplanted kidneys. At the level of genetic susceptibility, future studies should integrate genetic and epigenetic analyses to identify variants that confer heightened risk from heavy metal exposure in KTRs. Building polygenic risk scores that incorporate key polymorphisms in metal-handling and antioxidant pathways could enable more precise stratification of patients and personalized management strategies. At the clinical translation level, the critical task is to design rigorous intervention trials to validate novel therapeutic strategies that target antioxidant defense, maintain mitochondrial function, and modulate the effects of probiotics. The feasibility of incorporating environmental exposure control into comprehensive post-transplant management should be evaluated, ultimately translating research findings into clinical guidelines and public health policies that effectively enhance graft long-term survival.

## 7 Conclusions

This review systematically elucidates that environmental and occupational heavy metal exposure constitutes a significant threat to the long-term prognosis of KTRs. Through accumulation in the renal cortex, induction of mitochondrial dysfunction, and catalysis of Fenton reactions, these metals trigger bursts of reactive oxygen species that disrupt intracellular redox homeostasis. This cascade activates inflammatory signaling pathways, promotes the progression of fibrosis, and induces apoptosis, ultimately accelerating the progression of chronic transplant kidney injury. Given the graft’s inherent susceptibility, immunosuppressed state, and potential impaired excretory function, these toxic effects are further amplified. Therefore, recognizing environmental heavy metal exposure as a key modifiable risk factor and incorporating targeted monitoring and protective strategies into clinical management are crucial for improving long-term graft survival.
